# Glycosylation of Residue 141 of Subtype H7 Influenza A Hemagglutinin (HA) Affects HA-Pseudovirus Infectivity and Sensitivity to Site A Neutralizing Antibodies

**DOI:** 10.1371/journal.pone.0149149

**Published:** 2016-02-10

**Authors:** Esmeralda Alvarado-Facundo, Russell Vassell, Falko Schmeisser, Jerry P. Weir, Carol D. Weiss, Wei Wang

**Affiliations:** 1 Laboratory of Immunoregulation, Division of Viral Products, Center for Biologics Evaluation and Research, US Food and Drug Administration, 10903 New Hampshire Avenue, Silver Spring, Maryland 20993, United States of America; 2 Laboratory of DNA Viruses, Division of Viral Products, Center for Biologics Evaluation and Research, US Food and Drug Administration, 10903 New Hampshire Avenue, Silver Spring, Maryland 20993, United States of America; Icahn School of Medicine at Mount Sinai, UNITED STATES

## Abstract

Human infections with H7 subtype influenza virus have been reported, including an H7N7 outbreak in Netherlands in 2003 and H7N9 infections in China in 2013. Previously, we reported murine monoclonal antibodies (mAbs) that recognize the antigenic site A of H7 hemagglutinin (HA). To better understand protective immunity of H7 vaccines and vaccine candidate selection, we used these mAbs to assess the antigenic relatedness among two H7 HA isolated from past human infections and determine residues that affect susceptibility to neutralization. We found that these mAbs neutralize pseudoviruses bearing HA of A/Shanghai/02/2013(H7N9), but not A/Netherlands/219/2003(H7N7). Glycosylation of the asparagine residue at position 141 (N141) (N133, H3 HA numbering) in the HA of A/Netherlands/219/2003 HA is responsible for this resistance, and it affects the infectivity of HA-pseudoviruses. The presence of threonine at position 143 (T135, H3 HA numbering) in the HA of A/Netherlands/219/2003, rather than an alanine found in the HA of A/Shanghai/02/2013(H7N9), accounts for these differences. These results demonstrate a key role for glycosylation of residue N141 in affecting H7 influenza HA-mediated entry and sensitivity to neutralizing antibodies, which have implications for candidate vaccine design.

## Introduction

Avian influenza viruses with potential to acquired human-to-human transmission are a public health concern. Human infections with H7 subtype influenza virus have been documented in people who had direct contact with infected birds or humans with influenza infection [1, 2]. More recently, a novel avian influenza virus A (H7N9) emerged in China in 2013 with about 36% fatality rate [3], although there is no evidence of sustained person-to-person spread [4]. Because of the concerns of potential future H7 pandemics, several candidate vaccine strains for the H7 subtype were developed before the 2013 H7N9 outbreak [5–7]. The immunogenicity of these earlier H7 vaccines was poor [8, 9]. More recently, candidate H7N9 vaccines derived from A/Shanghai/02/2013 have been prepared [10] and tested for immunogenicity in clinical trials [11–14]. However the protective immunity induced by H7 vaccines is not completely understood. The glycosylation of HA can influence its antigenicity and thus could play an important role in candidate vaccine strain selection and vaccination effectiveness. Different cell substrates can also affect glycosylation [15, 16]. It has been demonstrated that gain of a side-chain, resulting in an antigenic change by preventing antibody binding, can have a selective advantage [15, 16].

To better understand immunity to H7 influenza and vaccine candidate selection, we assessed antigenic relatedness among H7 HA. We found that glycosylation of an asparagine residue at position 141 (N133, H3 HA numbering) in hemagglutinin plays an important role in modifying the infectivity and neutralization sensitivity of pseudoviruses bearing HA from A/Shanghai/02/2013 and A/Netherlands/219/2003.

## Material and Methods

### Plasmids and cell lines

Full-length open reading frame (ORF) from A/Shanghai/02/2013 HA (GISAID accession # EPI439502) and A/Netherlands/219/2003 HA (GenBank accession # AY338459) were synthetized by GenScript (Piscataway, NJ) and placed into pCMV/R expression plasmid. Mutations in HA were introduced using standard molecular biology protocols and confirmed by sequencing. pCMV/R plasmid expressing influenza A/California/04/2009 neuraminidase (GenBank accession # FJ966084) was previously described [17]. The HIV gag/pol (pCMV ΔR8.2) and Luciferase reporter (pHR’CMV-Luc) plasmids described previously [18, 19] were obtained from Gary J. Nabel (NIH, Bethesda, MD). The codon-optimized HAT gene expression construct pCAGGS-HATcop (HATcop) was described previously [20]. 293T cells were cultured in Dulbecco’s modified eagle medium (DMEM) with high glucose, L-Glutamine, MEM non-essential amino acids, penicillin/streptomycin and 10% fetal calf serum.

### Antibodies

The mouse monoclonal antibodies (mAbs), 5A6, 4A2 and 2C4, against Influenza A/Shanghai/2/2013 HA were previously described [21]. The mouse mAb H9-A22 against A/Puerto Rico/8/34 HA [22] was obtained from Dr. Jonathan W. Yewdell (NIH, Bethesda, MD). Goat antisera against A/Netherlands/219/2003 HA were obtained from BEI resources (Manassas, VA). HIV-1 p24 Hybridoma (183-H12-5C) from Dr. Bruce Chesebro was obtained through the AIDS Research and Reference Reagent Program, Division of AIDS, NIAID, NIH.

### Production of HA-pseudoviruses

HA-pseudoviruses carrying a luciferase (Luc) reporter gene were produced in 293T cells as described previously [20]. Briefly, 1.0 μg of A/Shanghai/02/2013 HA or 1.0 μg of A/Netherlands/219/2003 HA, 3 μg of A/California/04/2009 neuraminidase, 5 μg of pCMV ΔR8.2, and 5 μg of pHR’CMV-Luc plasmids were cotransfected into 293T cells with FuGENE 6 (Promega, Madison, WI). For A/Shanghai/02/2013 HA-pseudovirus production, 1 μg of codon-optimized human airway trypsin-like protease (HATcop) were included in cotransfection. HA-pseudoviruses were collected 48 hr post transfection, filtered through a 0.45-μm low protein binding filter, and used immediately or stored at -80°C. HA-pseudovirus infectivity titers were determined by infecting 293T cells with HA-pseudoviruses for 48 hr prior to measuring luciferase activity in infected cells using luciferase assay reagent (Promega, Madison, WI), as described previously [20], and expressed as relative luminescence unit (RLU) per ml of HA-pseudovirus supernatants. Pseudoviruses were quantified by HIV-1 p24 gag ELISA assay (AIDS Vaccine Program, NCI-Frederick Cancer Research and Development Center, Frederick, MD), as described previously [23]. HA and HIV-1 p24 gag in pseudoviruses were measured by immunoblotting analysis using goat antisera against A/Netherlands/219/2003 HA and mouse mAb (183-H12-5C) against HIV-1 p24 Gag, respectively.

### Neutralization assay

HA-pseudoviruses containing approximately 15 ng/ml of p24 antigen and 12 ng/ml of HA were incubated with mAb samples for 1 hr at 37°C, prior to inoculating mixtures onto 293T cells, as described previously [20]. The mAb dilution causing a 95% reduction of luciferase activity compared to control (IC95), calculated using Graphpad Prism software, was used as the neutralization titer [20].

### Hemagglutination inhibition and ELISA assays

The hemagglutination inhibition assay (HI) was performed in 96-well plates (V-bottom) by a standard method, essentially as described in the WHO Manual on Animal Influenza Diagnosis and Surveillance (http://www.who.int/csr/resources/publications/influenza/en/whocdscsrncs20025rev.pdf) using 0.5% turkey red blood cells (Lampire Biological Laboratories, Pipersville, PA) suspended in PBS (pH 7.2). Standard ELISA assay was applied to detect the binding of H7 mAb to HA-pseudovirus. Briefly, HA-pseudoviruses were coated onto ELISA plates (10 μg/ml, 100 μl/well). The virus-coated plates were blocked and then incubated with mAb at different concentrations. After an additional incubation with peroxidase-conjugated goat anti-mouse IgG (KPL, Gaithersburg, MD), the signal was developed using TMB as substrate. The reaction was stopped with 1N H_2_SO_4_ and OD450 values were recorded.

### Deglycosylation of HA

To determine the occupancy by a glycan of aspartic acid in the predicted glycosylation site in HA, HA-pseudoviruses were digested with Peptide: N-Glycosidase F (PNGase F) kit (New England Biolabs, Ipswich, MA) according to the manufactured instructions. Briefly, HA-pseudoviruses made in serum-free media were pelleted by centrifugation for 3 hr at 13,000 x *g*, then re-suspended in phosphate-buffered saline (PBS) containing 1% NP40. HA-pseudoviruses were denatured with 1X Glycoprotein Denaturing Buffer at 100°C for 10 min. After the addition of G7 Reaction Buffer and PNGase F, the mixtures were incubated for 1hr at 37°C. Samples were resolved on SDS-PAGE, and detected by Western blot using Goat antisera against A/Netherlands/219/2003 HA.

### Immunoprecipitation

HA-pseudoviruses were pelleted by centrifugation for 3 hr at 13,000 x *g*, re-suspended in PBS containing 1% NP40 and 1% n-dodecyl β-D- maltoside (DDM), and incubated for 1hr at 37°C. HA-pseudovirus samples were mixed with anti-HA mAb, and incubated for 1hr at 37°C. Protein G magnetic beads were then added into the samples, and the samples were incubated overnight at 4°C. The Protein-G magnetic beads were washed for 10 min at 4°C for 4 times with PBS containing 1% NP40. The immunoprecipitation samples were resolved on SDS-PAGE and detected by Western blot using Goat antisera against A/Netherlands/219/2003 HA.

### Computational analysis

The HA structure comparison and residues locations on HA were analyzed by UCSF Chimera program (http://www.cgl.ucsf.edu/chimera/) using the Protein Data Bank (PDB) entries 4DJ6 [24] and 4LN6 [25].

### Data analysis

Infectivity data reported were from at least three independent experiments. T test for paired data comparison and corresponding P value were analyzed using GraphPad Prism software. P values <0.05 were considered statistically significant.

## Results

### 5A6, 4A2 and 2C4 mAbs neutralize A/Shanghai/02/2013, but not A/Netherlands/219/2003

We previously described a panel of H7 monoclonal antibodies (mAbs) (5A6, 4A2 and 2C4) that recognize antigenic site A in the HA of A/Shanghai/02/2013(H7N9) [21], which is structurally similar to H3 HA. Because this site is conserved across H7 strains, it is a potentially important protective epitope for cross-reactive antibodies. We compared the neutralizing activity of these mAbs against pseudoviruses bearing HA of A/Netherlands/219/2003 and A/Shanghai/02/2013, two H7 viruses isolated from human infections of H7N7 in 2003 [26] and H7N9 in 2013 [27]. Both viruses are Eurasian lineage viruses and are similar in sequence, differing at only 11 amino acids in the mature HA1 portion of the hemagglutinin (excluding the polybasic cleavage site at the junction of HA1 and HA2 in H7N7). Despite the conservation of amino acids in site A, we found that these mAbs (5A6, 4A2 and 2C4) only neutralized A/Shanghai/02/2013(H7N9) HA-pseudoviruses, and not A/Netherlands/219/2003(H7N7) HA-pseudoviruses. As expected, the A/Puerto Rico/8/1934(H1N1) HA-pseudovirus was also not neutralized by the H7 mAbs (Table 1).

**Table 1:** Neutralization titers of H7 Monoclonal Antibodies (mAbs).

Virus	Neutralization Titer (IC95) (μg/ml)			
	5A6*	4A2*	2C4*	H9-A22**
A/Shanghai/02/2013	2.69	0.54	0.80	>10
A/Shanghai/02/2013 A143T	>10	>10	>10	>10
A/Shanghai/02/2013 R149G	>10	>10	>10	>10
A/Shanghai/02/2013 S183D	0.95	0.38	0.64	>10
A/Shanghai/02/2013 V195G	0.93	0.45	0.35	>10
A/Shanghai/02/2013 A198T	3.49	0.60	0.72	>10
A/Netherlands/219/2003	>10	>10	>10	>10
A/Netherlands/219/2003 T143A	2.42	0.90	0.93	>10
A/Netherlands/219/2003 T143A, R149G	>10	>10	>10	>10
A/Netherlands/219/2003 D183S	>10	>10	>10	>10
A/Netherlands/219/2003 G195V	>10	>10	>10	>10
A/Netherlands/219/2003 T198V	>10	>10	>10	>10
A/Puerto Rico/8/1934	>10	>10	>10	0.15

* mAbs 5A6, 4A2, and 2C4 to site A were raised against HA of A/Shanghai/02/2013.

** mAb were raised against A/Puerto Rico/8/1934.

Similarly, these mAbs (5A6, 4A2 and 2C4) only inhibited A/Shanghai/02/2013(H7N9), but not A/Netherlands/219/2003(H7N7), HA-pseudovirus-mediated hemagglutination (Table 2).

**Table 2:** Hemagglutination inhibition titers of H7 Monoclonal Antibodies (mAbs).

Virus	Hemagglutination Inhibition Titers (μg/ml)^a^			
	5A6*	4A2*	2C4*	H9-A22**
A/Shanghai/02/2013	12.5	12.5	12.5	>100
A/Shanghai/02/2013 A143T	>100	>100	>100	>100
A/Shanghai/02/2013 S183D	6.25	6.25	6.25	>100
A/Shanghai/02/2013 V195G	6.25	6.25	6.25	>100
A/Shanghai/02/2013 A198T	12.5	12.5	12.5	>100
A/Netherlands/219/2003	>100	>100	>100	>100
A/Netherlands/219/2003 T143A	6.25	12.5	6.25	>100
A/Netherlands/219/2003 D183S	>100	>100	>100	>100
A/Netherlands/219/2003 G195V	>100	>100	>100	>100
A/Netherlands/219/2003 T198V	>100	>100	>100	>100
A/Puerto Rico/8/1934	>100	>100	>100	3.13

^a^ Lowest antibody concentration that inhibited H7 virus-mediated hemagglutination of turkey red blood cells; initial mAb concentration 100 μg/ml.

* mAbs 5A6, 4A2, and 2C4 to site A were raised against HA of A/Shanghai/02/2013.

** mAb were raised against A/Puerto Rico/8/1934.

### HA residue 143, beyond antigenic site A, modifies viral sensitivity to neutralizing mAbs

**Fig 1:**
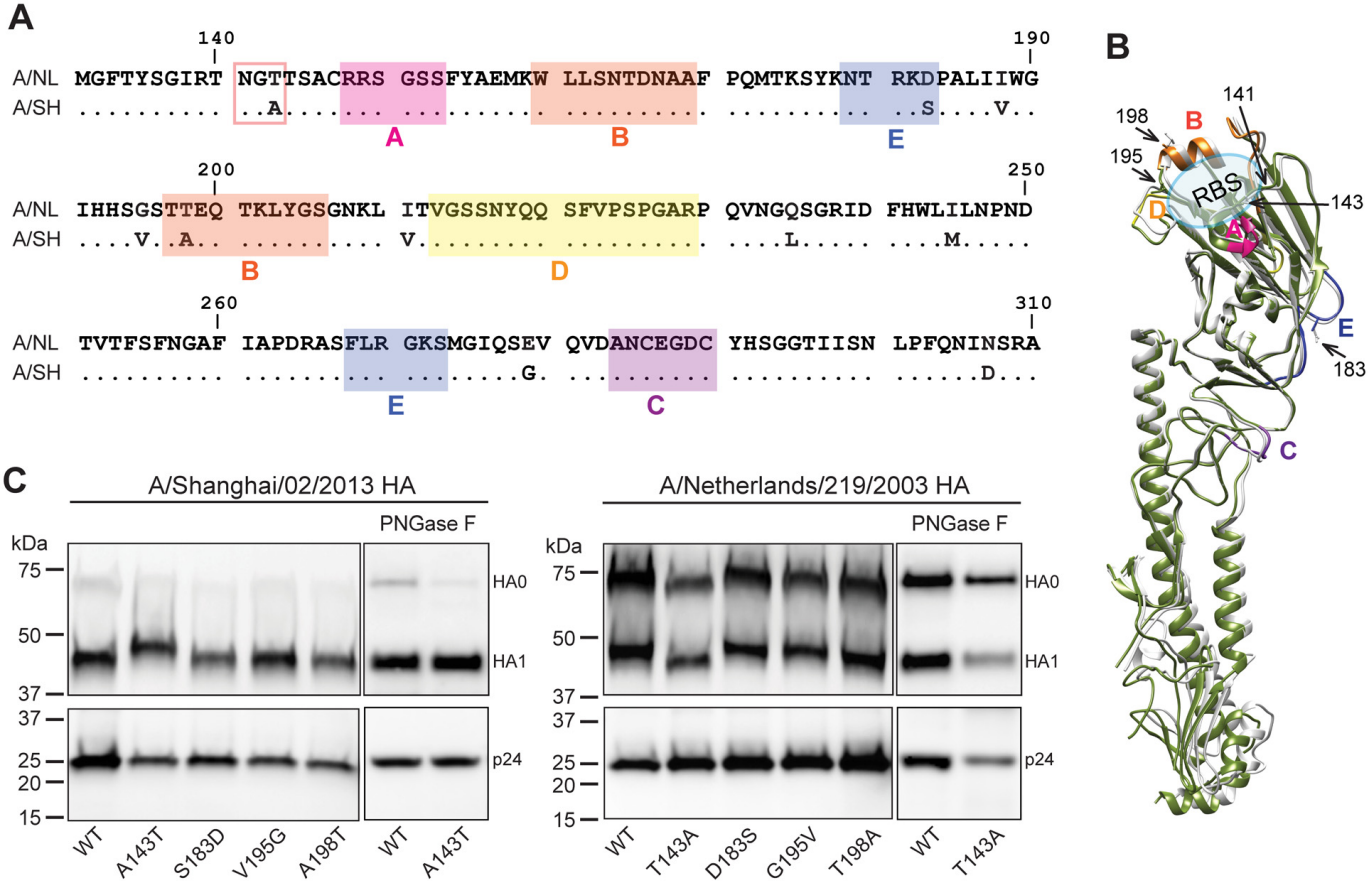
Comparison of A/Shanghai/02/2013 and A/Netherlands/219/2003 HA. **(A)** Alignment of A/Shanghai/02/2013 and A/Netherlands/219/2003 HA amino acid residues 130 to 310. Predicted antigenic sites A (pink), B (orange), C (purple), D (yellow), and E (blue) are highlighted. Glycosylation site N141 is marked in a square. **(B)** Overlapping of A/Netherlands/219/2003 HA (grey, PDB: 4DJ6) and A/Shanghai/02/2013 HA (green, PDB: 4LN6) crystal structures. Only monomers are shown. Predicted antigenic sites A (pink), B (orange), C (purple), D (yellow), and E (blue) are labeled. RBS: receptor binding site. **(C)** Western blot comparison of A/Shanghai/02/2013 and A/Netherlands/219/2003 HA treated with and without PNGase F. Wild type (WT) HA and its mutants were detected with goat antisera against A/Netherlands/219/2003 HA (Top panel). The p24 in HA-pseudoviruses, which served as loading controls, were detected with mouse mAb (183-H12-5C) against HIV-1 p24 Gag (Bottom panel). Data shown are representative of two independent experiments.

The amino acid sequences of HA show that all antigenic sites, except sites B and E, are identical between A/Shanghai/02/2013 and A/Netherlands/219/2003 (Fig 1A). In sites B and E, respectively, A/Shanghai/02/2013 HA has serine at residue183 (S183) (S174, H3 HA numbering) and an alanine at residue198 (A198) (A189, H3 HA numbering), while A/Netherlands/219/2003 HA has aspartic acid at residue183 (D183) and a threonine at residue 198 (T198). In addition, a few other residues differ near the antigenic sites (Fig 1A). Because changes in the binding affinity of HA for its receptor sialic acid could alter the potency of neutralizing antibodies [22, 28–31], we focused on amino acid differences at residues 143 (135, H3 HA numbering) and 195 (186, H3 HA numbering) around the receptor binding site (RBS) (Fig 1B). A/Netherlands/219/2003 HA has a threonine at residue 143 (T143) (T135, H3 HA numbering) and a glycine at residue195 (G195) (G186, H3 HA numbering), while A/Shanghai/02/2013 HA has an alanine (A143) (A135, H3 HA numbering) and valine (V195) (V186, H3 HA numbering) in these RBS positions.

To determine whether T143 or G195 is responsible for A/Netherlands/219/2003 resistance to neutralizing mAbs that target antigenic site A, we introduced mutations at these positions into A/Shanghai/02/2013 HA and A/Netherlands/219/2003 HA. In both neutralization and hemagglutination inhibition assays, we found that A/Shanghai/02/2013 HA-pseudoviruses with an alanine to threonine substitution at position 143 (A143T) in HA became resistant to all mAbs in our panel, while A/Netherlands/219/2003 HA-pseudoviruses bearing a threonine to alanine substitution at position 143 (T143A) in HA gained sensitivity to these neutralizing mAbs (Tables 1 and 2). Notably, neutralization sensitivity was not changed by other substitutions, including a serine to aspartic acid substitution at position 183 (S183D), a valine to glycine substitution at position 195 (V195G), and an alanine to threonine substitution at position 198 (A198T) in A/Shanghai/02/2013 HA or an aspartic acid to serine substitution at position 183 (D183S), a glycine to valine substitution at position 195 (G195V), and a threonine to alanine substitution at position 198 (T198A) in A/Netherlands/219/2003 HA (Tables 1 and 2). These results indicate that T143 alone is responsible for resistance of A/Netherlands/219/2003 to neutralizing mAbs that target antigenic site A.

Previously, we generated an escape mutant virus with an arginine to glycine substitution at position 149 (R149G) in A/Shanghai/02/2013 HA that confers resistance to these neutralizing mAbs [21] (Table 1). To extend these findings, we combined T143A and R149G in A/Netherlands/219/2003 HA. The A/Netherlands/219/2003 HA-pseudoviruses with T143A and R149G in HA became resistant to neutralizing mAbs (Table 1), confirming that these mAbs target antigenic site A.

### Glycosylation of asparagine 141 is occupied in A/Netherlands/219/2003 HA

To understand the resistance mechanism conferred by T143, we analyzed the HA sequence and found that T143 in A/Netherlands/219/2003 HA creates a glycosylation motif (N-X-T/S) involving asparagine 141 (N141) (N133, H3 numbering). Western blot analysis showed that A/Shanghai/02/2013 HA containing A143T has a higher molecular weight than wild type A/Shanghai/02/2013 HA (Fig 1C). Similarly, the wild-type A/Netherlands/219/2003 HA with T143 has a higher molecular weight than A/Netherlands/219/2003 HA with T143A. Moreover, PNGase F treatment, which removes the N-linked glycans, resulted in a decrease of the molecular weight of both A/Shanghai/02/2013 HA with A143T and wild type A/Netherlands/219/2003 HA that contains T143 (Fig 1C). These results suggest that N141 is glycosylated when a threonine is present at position 143 (T143) in the H7 HA, and thus glycosylation of N141 is responsible for the lack of sensitivity of A/Netherlands/219/2003 to neutralizing mAbs that target to antigenic site A. The more efficient processing of HA0 in A/Shanghai/02/2013 HA-pseudoviruses compared to A/Netherlands/219/2003 HA-pseudoviruses (Fig 1C) is due to the addition a protease (HAT) expression vector during the preparation of A/Shanghai/02/2013 HA-pseudoviruses. Because A/Netherlands/219/2003 HA contains a polybasic sequence that can be cleaved during biogenesis by cellular proteases such as furin, a protease expression vector is not used to produce HA-pseudoviruses for strains with the polybasic cleavage site.

### Glycosylation prevents binding of neutralizing mAbs to antigenic site A

**Fig 2:**
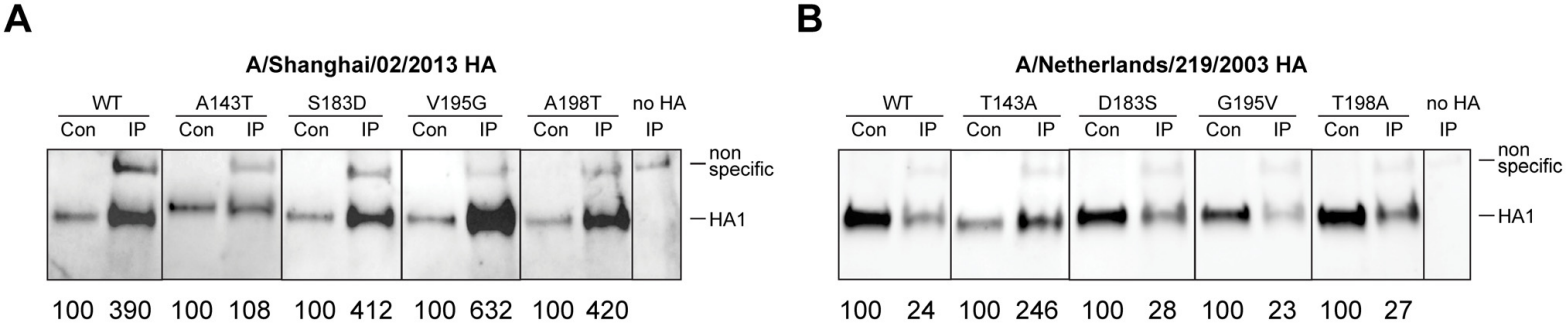
Threonine 143 in HA blocks neutralizing antibodies binding to antigenic site. Immunoprecipitation of A/Shanghai/02/2013 HA (A) and A/Netherlands/219/2003 HA (B) with mAb 5A6. The relative levels of HA shown by Western blots were detected with goat antisera against A/Netherlands/219/2003 HA. Percentages of the HA signal intensities, normalized to the 1/10 volume of the input used for the immunoprecipitation (control), are indicated below each blot. Con: control; IP: immunoprecipitation. Data shown are representative of two independent experiments.

We next investigated the mechanism of resistance to neutralization conferred by glycosylation of N141. In immunoprecipitation assays using HA-pseudoviruses, we found that the A143T substitution reduced mAb 5A6 binding to A/Shanghai/02/2013 HA approximately four fold, while the S183D, V195G and A198T substitutions did not decrease binding (Fig 2A). As a specificity control, we did not observe mAb 5A6 binding to H1, H3 and H5 HAs (data not shown). Similarly, the T143A substitution, but not D183S, G195V, or T198A substitutions, enhanced A/Netherlands/219/2003 HA binding to mAb 5A6 approximately ten fold (Fig 2B). These results demonstrate that glycosylation of N141 directly blocks the interaction of the neutralizing mAbs with H7 HA.

To further confirm this finding, the binding of H7 mAbs to H7 HA was also evaluated using an ELISA assy. The ELISA results showed that the A143T substitution reduced binding of mAbs 5A6, 4A2 and 2C4 to A/Shanghai/02/2013 HA-pseudoviruses approximately ten fold, while the S183D, V195G and A198T substitutions did not decrease binding (Table 3). As a specificity control, these mAbs did not bind to the A/Puerto Rico/8/1934 (H1N1) HA-pseudovirus, while the mAb H9-A22, which is specific for this strain, did bind this HA-pseudovirus. Similarly, the T143A substitution, but not D183S, G195V, or T198A substitutions, enhanced binding of mAbs 5A6, 4A2 and 2C4 to A/Netherlands/219/2003 HA-pseudoviruses approximately ten to twenty fold (Table 3).

**Table 3:** ELISA end-point titers of H7 Monoclonal Antibodies (mAbs).

Virus	ELISA End-point Titers (μg/ml)^a^			
	5A6*	4A2*	2C4*	H9-A22**
A/Shanghai/02/2013	0.02	0.04	0.02	>4
A/Shanghai/02/2013 A143T	0.2	0.4	0.2	>4
A/Shanghai/02/2013 S183D	0.02	0.04	0.02	>4
A/Shanghai/02/2013 V195G	0.01	0.02	0.01	>4
A/Shanghai/02/2013 A198T	0.02	0.04	0.02	>4
A/Netherlands/219/2003	0.4	0.4	0.4	>4
A/Netherlands/219/2003 T143A	0.02	0.04	0.02	>4
A/Netherlands/219/2003 D183S	0.4	0.4	0.4	>4
A/Netherlands/219/2003 G195V	0.4	0.4	0.4	>4
A/Netherlands/219/2003 T198V	0.4	0.4	0.4	>4
A/Puerto Rico/8/1934	>4	>4	>4	0.001

^a^ The end-point titer was defined as the lowest antibody concentration that gave an absorbance value greater than 0.05 at 450 nm.

* mAbs 5A6, 4A2, and 2C4 to site A were raised against HA of A/Shanghai/02/2013.

** mAb were raised against A/Puerto Rico/8/1934.

### N141 glycosylation of HA decreases viral infectivity

**Fig 3:**
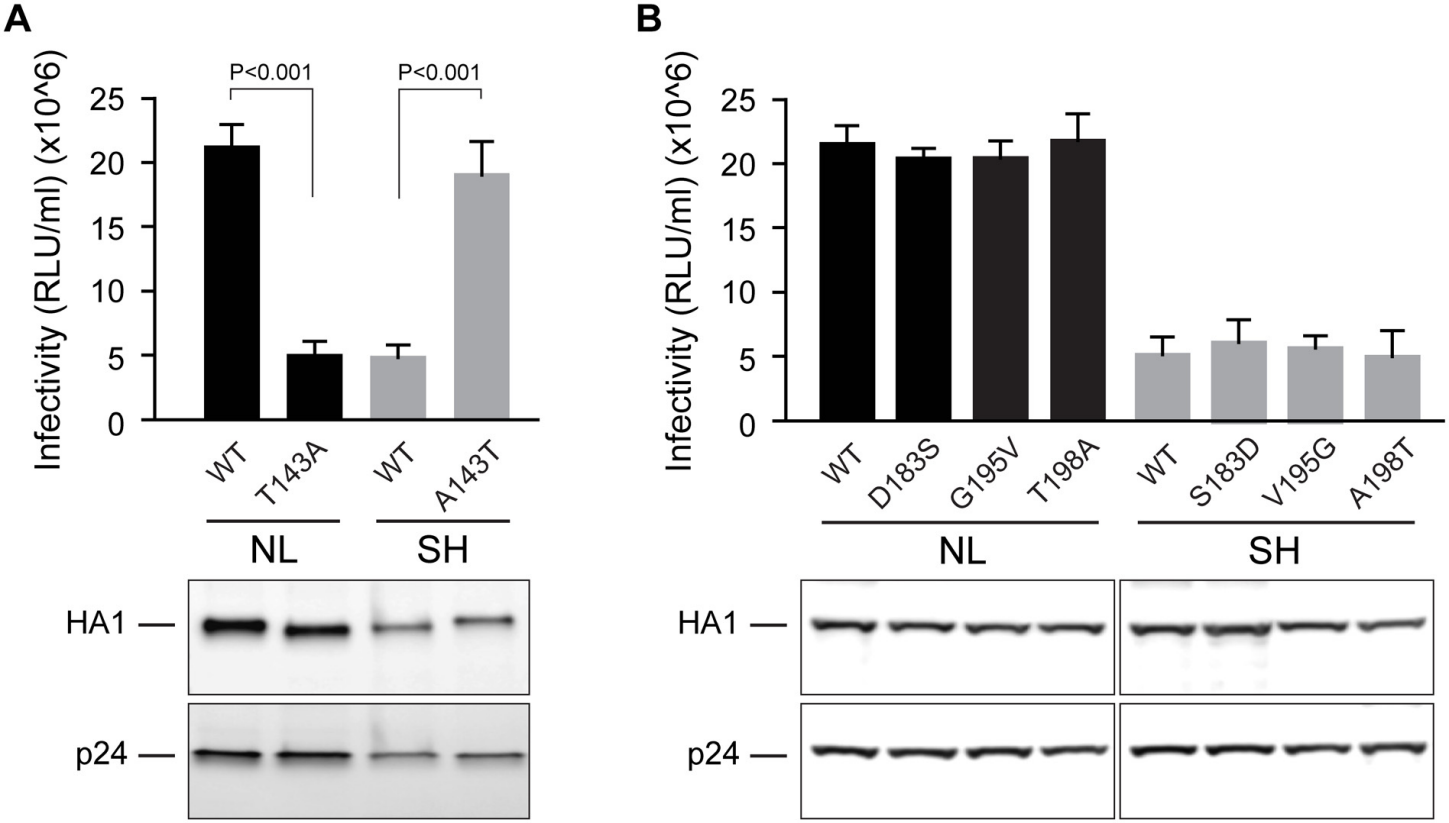
Threonine 143 in HA enhances H7 pseudovirus infectivity. **(A)** Comparison of WT and mutation on residue 143. **(B)** Comparison of WT and mutations on residues 183, 195 and 198. The infectivity of pseudoviruses bearing wild type (WT) HA and its mutants is compared (Top panels in **A** and **B**). Data are shown as mean and standard deviation of three independent experiments. P<0.001 of T-test were shown. The levels of mature HA (HA1) and p24 in HA-pseudoviruses shown by Western blotting were detected with goat antisera against A/Netherlands/219/2003 HA and mouse mAb (183-H12-5C) against HIV-1 p24 Gag, respectively (Bottom panels in **A** and **B**). NL: A/Netherlands/219/2003 HA-pseudoviruses; SH: A/Shanghai/02/2013 HA-pseudoviruses.

We noticed that wild type A/Shanghai/02/2013 HA-pseudoviruses displayed a lower level of infectivity than wild type A/Netherlands/219/2003 HA-pseudoviruses. Since N141 is near the RBS (Fig 1B), we also assessed whether glycosylation of N141 affects HA binding to receptors and thus pseudovirus infectivity. The infectivity of HA-pseudoviruses showed that only A143T (Fig 3A), but not S183D, V195G, and A198T substitutions (Fig 3B), improved A/Shanghai/02/2013 HA-pseudovirus infectivity. Likewise, A/Netherlands/219/2003 HA-pseudoviruses with a T143A substitution (Fig 3A), but not D183S, G195V, or T198A substitutions (Fig 3B), reduced A/Netherlands/219/2003 infectivity. Thus, glycosylation of residue 143 in both H7 subtype HA affects pseudovirus infectivity, as well as susceptibility to neutralization.

## Discussion

Candidate H7 vaccines have been made to prepare for potential future H7 pandemics, but the immunogenicity of these vaccines was generally poor [8, 9], and the protective immunity induced by the H7 vaccines is not completely understood. To better understand immune responses to H7 subtypes, we assessed antigenic relatedness of two H7 HA isolated from human outbreaks and found that glycosylation of N141 (N133, H3 HA numbering) has a significant impact on H7 HA-pseudovirus infectivity and sensitivity to neutralizing antibodies that target antigenic site A on HA.

The effects of HA glycosylation on HA antigenicity have been well documented. Gain of a side-chain can prevent antibody binding and lead to antigenic changes [15, 16]. HA glycosylation may provide a selective advantage for influenza virus to escape immune-protection [15, 16, 32–35]. Thus, understanding HA glycosylation may be important for selection of candidate vaccine strains, as well as how propagation of vaccine viruses in different cell substrates may affect immunogenicity [15, 16]. We previously found that the conserved antigenic site A in H7 HA is a potentially important protective epitope for cross-reactive antibody responses [21]. Our finding that glycosylation of N141, which occurs naturally in the A/Netherlands/219/2003 virus, protects H7 virus from neutralization by site A antibodies again suggests the importance of antigenic site A.

The candidate H7 vaccine strains available from WHO [10, 36], including recent H7N9 candidate vaccines derived from A/Shanghai/2/213 and A/Anhui/1/2013, as well as older Eurasian H7 viruses derived from A/mallard/Netherlands/12/2000, do not have the glycosylation site at N141. This suggests that an antibody response to antigenic site A elicited by vaccines developed from these candidate vaccine viruses may not contribute to protection against H7 viruses that are glycosylated at N141. While the protective antibody response likely involves multiple HA epitopes, this observation emphasizes the importance of a better understanding of the nature of a protective antibody response to H7 influenza viruses. Similarly, a more complete understanding of the antibody response to candidate vaccines is needed. Interestingly, T143A in A/Netherlands/219/2003 HA was also recently shown to improve the immunogenicity of A/Netherlands/219/2003 HA [37], further highlighting the role of N141 glycosylation in masking site A of H7 HA.

Unexpectedly, we also found that N141 glycosylation affects pseudovirus infectivity. Both A/Netherlands/219/2003 and A/Shanghai/02/2013 HA-pseudoviruses displayed higher infectivity when residue at 143 was glycosylated. This finding is consistent with previous reports of T143 in A/Netherlands/219/2003 HA being associated with enhanced viral replication [38] and of an A143T substitution in A/Anhui/1/2013 (H7N9) HA emerging during transmission in ferret [39]. Altogether, our data, along with other reports, indicate that glycosylation of N141 in HA plays an important role in H7 viral infectivity and sensitivity to neutralization and should be taken into consideration when selecting strains for H7 vaccines.
